# Mechanisims of asthma and allergic disease – 1087. Serine protease activity of per a 10 is requried to activiate airway epithelial cells

**DOI:** 10.1186/1939-4551-6-S1-P83

**Published:** 2013-04-23

**Authors:** Sagar Laxman Kale, Naveen Arora

**Affiliations:** 1 Allergy and Immunology, Institute of Genomics and Integrative Biology, Delhi, India

## Background

Airway epithelial cells play an important role in initiating airway allergic responses by secreting a variety of proinflammatory cytokines. The present study was aimed to elucidate the effect of serine protease activity of Per a 10 on biochemical properties of an airway epithelial cell line.

## Methods

Alveolar type II epithelial cell line A549 was incubated with active (nPer a 10), AEBSF inactivated (iPer a 10), heat inactivated (ΔPer a 10) native Per a 10 and inactive recombinant Per a 10 (rPer a 10). After incubation pro-inflammatory cytokines IL-6, IL-8 and GMCSF were measured by ELISA. Intracellular Ca^2+^ mobilisation was determined in A549 cells by Fluo 4AM. PAR-2 derived peptide (GTNRSSKGRSLIGKVDGTSHVTGKGVTC) was incubated with nPer a 10 and resultant cleaved products were subjected to LC-MS.

## Results

nPer a 10 caused desquamation of cells that led to the secretion of pro-inflammatory cytokines from A549 cells in concentration dependent manner. ΔPer a 10 whose activity was abolished by heat treatment and rPer a 10 failed to secrete any of these cytokines. iPer a 10 showed some residual activity after treating with AEBSF and caused lower but significant secretion of proinflammatory cytokines. Per a 10 was able to mobilise Ca^2+^ in a concentration and activity dependent manner. nPer a 10 cleaved the PAR-2 derived peptide (GTNRSSKGRSLIGKVDGTSHVTGKGVTC) between arginine and serine residues as determined by LC-MS, to expose the PAR-2 specific ligand SLIGKV. Whereas rPer a 10 and inactive Per a 10 failed both to mobilise Ca^2+^ and to cleave PAR-2 derived peptide. Thus Per a 10 activated epithelial cells to secrete proinflammatory cytokines in activity dependent manner via PAR-2 receptors.

## Conclusions

Per a 10 activates epithelial cells to secrete proinflammatory cytokines that mediate the allergic responses and the activation is through the cleavage of PAR-2 receptors.

